# Complex Genetic Interactions between Piwi and HP1a in the Repression of Transposable Elements and Tissue-Specific Genes in the Ovarian Germline

**DOI:** 10.3390/ijms222413430

**Published:** 2021-12-14

**Authors:** Artem A. Ilyin, Anastasia D. Stolyarenko, Nikolay Zenkin, Mikhail S. Klenov

**Affiliations:** 1 Department of Molecular Genetics of the Cell, Institute of Molecular Genetics of National Research Centre «Kurchatov Institute», 2 Kurchatov Sq., 123182 Moscow, Russia; ilyin@img.ras.ru (A.A.I.); stol@img.ras.ru (A.D.S.); 2 Laboratory of Mechanisms of Replication of Damaged DNA, Institute of Molecular Genetics of National Research Centre «Kurchatov Institute», 2 Kurchatov Sq., 123182 Moscow, Russia; 3 Centre for Bacterial Cell Biology, Biosciences Institute, Faculty of Medical Sciences, Newcastle University, Baddiley-Clark Building, Richardson Road, Newcastle Upon Tyne NE2 4AX, UK; nikolay.zenkin@newcastle.ac.uk

**Keywords:** HP1, Piwi, piRNA, transposable elements, germline

## Abstract

Insertions of transposable elements (TEs) in eukaryotic genomes are usually associated with repressive chromatin, which spreads to neighbouring genomic sequences. In ovaries of *Drosophila melanogaster*, the Piwi-piRNA pathway plays a key role in the transcriptional silencing of TEs considered to be exerted mostly through the establishment of H3K9me3 histone marks recruiting Heterochromatin Protein 1a (HP1a). Here, using RNA-seq, we investigated the expression of TEs and the adjacent genomic regions upon Piwi and HP1a germline knockdowns sharing a similar genetic background. We found that the depletion of Piwi and HP1a led to the derepression of only partially overlapping TE sets. Several TEs were silenced predominantly by HP1a, whereas the upregulation of some other TEs was more pronounced upon Piwi knockdown and, surprisingly, was diminished upon a Piwi/HP1a double-knockdown. We revealed that HP1a loss influenced the expression of thousands of protein-coding genes mostly not adjacent to TE insertions and, in particular, downregulated a putative transcriptional factor required for TE activation. Nevertheless, our results indicate that Piwi and HP1a cooperatively exert repressive effects on the transcription of euchromatic loci flanking the insertions of some Piwi-regulated TEs. We suggest that this mechanism controls the silencing of a small set of TE-adjacent tissue-specific genes, preventing their inappropriate expression in ovaries.

## 1. Introduction

The highly conserved Heterochromatin Protein 1a (HP1a) plays a key role in gene silencing from fission yeast to humans through the formation and maintenance of repressive chromatin (for review, see Reference [1]). HP1a binds specifically to di- and trimethylated H3K9 histones (H3K9me2 and H3K9me3) by its chromodomain [2,3], inducing chromatin compaction and transcriptional repression [4,5,6,7,8,9]. Recent findings indicate the HP1a capability to aggregate into liquid droplets, promoting the phase separation of heterochromatin [6,10]. In *Drosophila melanogaster*, HP1a, also known as Su(var)205, defines large pericentromeric and telomeric heterochromatin domains but also interacts with many sites in euchromatin, including insertions of transposable elements (TEs) and protein-coding genes [11,12,13,14,15]. 

Repressive chromatin marks are necessary for TE silencing [16,17]. Insertions of TEs in euchromatin serve as local islands of silent chromatin that can spread to the nearby genomic regions up to several tens of kilobases and thereby affect the expression of adjacent host genes [15,18,19,20]. TEs usually make up large fractions of eukaryotic genomes, for example, approximately 40% of the human genome [21]. Despite the fact that TEs occupy a smaller part of the *Drosophila melanogaster* genome, they are extremely diverse in this organism, belonging to more than 120 families constituting three main groups: LINE and LTR retrotransposons and DNA transposons (for review, see References [22,23]). Different TE families are characterised by distinct temporal and spatial patterns of expression and transposition activity, which, in turn, can determine the differences in the mechanisms aimed at their regulation [24,25,26,27,28]. TEs tend to be especially active in gonadal cells, which is required for the implementation of their biological strategy to infect the genomes of gametes and thereby transfer new TE copies to the next generation of host organisms. Hence, a strong control over TEs by cellular defence systems ensures germline genome stability. 

In *Drosophila* ovaries, the piRNA pathway is known to induce both the transcriptional and posttranscriptional silencing of TEs [29,30,31]. In the course of transcriptional repression, the nuclear protein Piwi loaded with piRNAs recognises nascent TE transcripts, which leads to the establishment of chromatin silencing at the corresponding genome loci by deposition of the H3K9me2/3 repressive marks and subsequent binding of HP1a [20,32,33,34,35,36]. Besides, Piwi-piRNA complexes can trigger repression by recruiting H1 histone [37], transcriptional repressor Mael [20,37,38,39], reducing the active chromatin mark H3K4me2 [40,41] and other mechanisms. According to current models, Piwi’s interaction with nascent RNAs initiates the formation of molecular condensates based on the SFiNX/Pandas/PICTS/PNNP complex, which recruits different chromatin factors to induce silencing of the underlying DNA locus [42,43,44,45,46,47]. In this process, HP1a acts as a downstream effector of repression induced by Piwi, while one work shows that HP1a may also be required for the biogenesis of piRNAs corresponding predominantly to telomeric TEs [48]. Generally, the role of HP1a in the repression of TEs remains not well understood, especially taking into account that, in ovarian somatic cells, HP1a targets many TEs, which are not regulated by the piRNA pathway [14,49]. Another poorly studied aspect is the contribution of epigenetic silencing of TEs to host gene regulation. Although it has been shown that TEs silenced by Piwi can affect the expression of adjacent genes by the spreading of repressive chromatin in cultured ovarian somatic cells (OSC) [20], it remains unknown how widespread such regulation is in vivo and, in particular, in germ cells.

Here, we study the genetic interactions between Piwi and HP1a in the silencing of TEs and host gene regulation in ovarian germ cells. We revealed that particular TE families responded differently to Piwi, HP1a and Piwi/HP1a double germline-specific knockdowns, suggesting various modes of TE repression in the germline. By identifying TE integration sites in the analysed *Drosophila* line, we show that the loss of HP1a leads to the elevated transcription of euchromatic sequences flanking TE insertions, including those corresponding to Piwi-sensitive TEs. We found that a small set of protein-coding genes is upregulated due to this abolishment of the repressive influence from the nearby insertions of TEs. Interestingly, under normal conditions, these target genes are characterised by extremely low expression in ovaries while being capable of achieving high transcription levels in some other tissues. This finding suggests that Piwi-guided repression of TE chromatin may be involved in the control of tissue specificity of gene expression.

## 2. Results

### 2.1. Sets of TEs Upregulated upon Germline Knockdowns of Piwi and HP1a Overlap Only Partially

**Figure 1:**
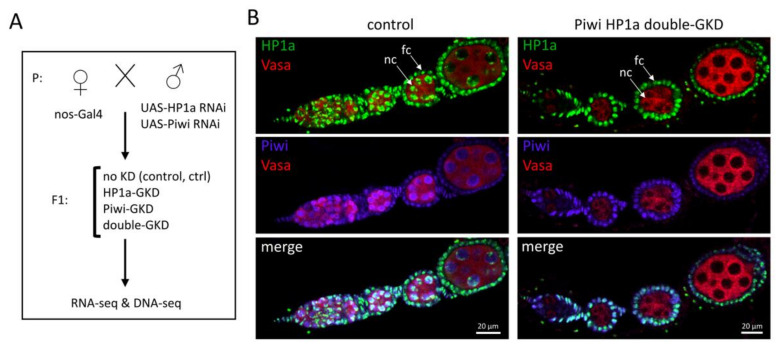
Generation of Piwi and HP1a separate and double-germline knockdowns. (**A**) Experimental design of the study. Control (no KD) flies and individuals with *nos-GAL4*-driven germline knockdowns (GKD) of Piwi and HP1a and the Piwi/HP1a double-GKD were obtained simultaneously from the same cross. (**B**) Control and double-GKD ovarioles immunostained for HP1a (green), Piwi (purple) and germ cell marker Vasa (red). Examples of germline nurse cells and somatic follicle cells are indicated as “nc” and “fc”, respectively.

To compare the effects of HP1a and Piwi on the expression of TEs, we performed *nos-GAL4*-driven germline-specific knockdowns (GKD) of Piwi and HP1a separately, as well as Piwi HP1a double-GKD. Since the localisation and number of insertions corresponding to different TE families can vary greatly between *Drosophila* lines, we took advantage of a crossing scheme in which the four analysed genotypes (hereafter, Ctrl, Piwi-GKD, HP1a-GKD and double-GKD) were obtained simultaneously as siblings from the same cross (Figure 1A). Therefore, they had similar genetic backgrounds, with the exception of one chromosome 2 and one chromosome 3 with Piwi RNAi and HP1a RNAi constructs, respectively.

Consistent with the previous reports [50,51], all the GKD females were sterile, and the HP1a-GKD individuals displayed much more severe defects of oogenesis than Piwi-GKD ones. HP1a-GKD females obtained from the cross at 25 °C had ovaries mostly devoid of germ cells, which was similar to previous findings [51]. However, we managed to achieve a less defective ovarian phenotype of HP1a-GKD ovaries by setting crosses at 18 °C. Under these conditions, most HP1a-GKD ovarioles successfully developed until oogenesis stage 7 harbouring egg chambers with the surviving germ cells, which were visualised by immunostaining for the Vasa germline marker (Figure 1B). Double-GKD ovaries were morphologically similar to HP1a-GKD ovaries. Immunostaining revealed the complete loss of Piwi and HP1a in the germ cells of the corresponding GKD ovaries (Figure 1B). Thus, the knockdowns were effective at 18 °C, whereas oogenesis at 25 °C was compromised likely due to the sensitivity of the HP1a-depleted germ cells to temperature conditions. We then performed a deep sequencing of rRNA-depleted total RNA (RNA-seq) isolated from the ovaries of Ctrl, Piwi-GKD, HP1a-GKD and double-GKD flies obtained at 18 °C. To minimise the morphological differences between these individuals, we dissected ovaries from new-born (0- to 1-day-old) females, since, at this time point, they were phenotypically most similar. In addition, we conducted whole-genome sequencing of the DNA of double-GKD flies to identify TE insertion sites in the corresponding genomes.

**Figure 2:**
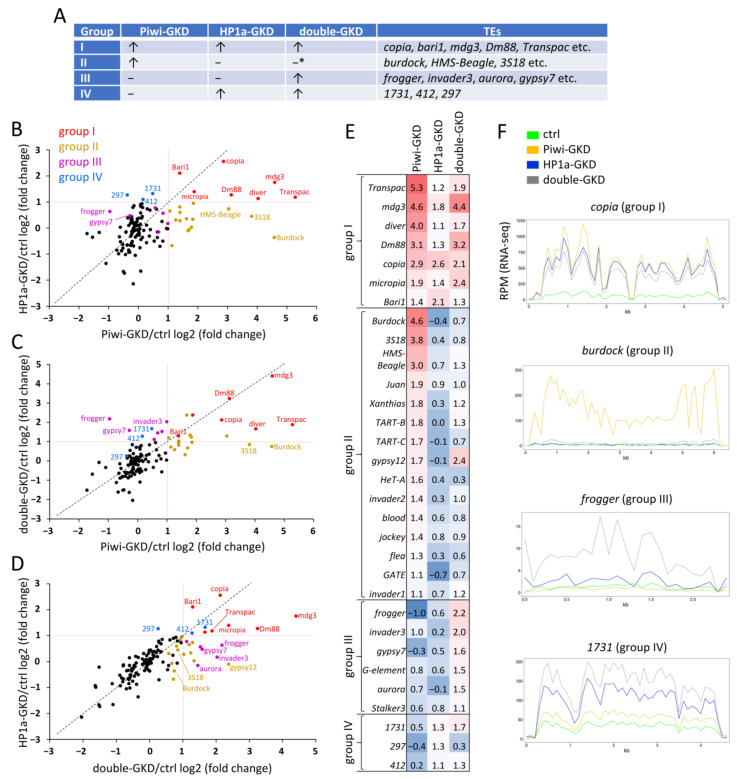
Germline knockdowns of Piwi and HP1a lead to the derepression of only partially overlapping TE sets. (**A**) Examples of TEs belonging to different groups according to their responses to Piwi-, HP1a- and double-GKD. Upregulation is indicated by arrows. * Several TEs of group II are upregulated upon double-GKD. (**B**) Scatterplot displaying fold upregulation (log2) upon Piwi-GKD relative to the Ctrl for TEs (*n* = 123) versus fold upregulation (log2) upon HP1a-GKD. The TEs of group I are marked in red, group II in dark yellow, group III in purple and group IV in blue. (**C**) The same for Piwi-GKD versus double-GKD. (**D**) The same for double-GKD versus HP1a-GKD. (**E**) Heatmap showing log2-fold upregulation upon Piwi-GKD, HP1a-GKD and double-GKD relative to the Ctrl for TEs belonging to different groups. Upregulation over 2-fold is shown in white and red and less than 2-fold (log2 = 1) is shown in blue. (**F**) Density profiles of the RNA-seq signal in 100-bp windows on *copia* (group I), *burdock* (group II), *frogger* (group III) and *1731* (group IV) TEs showing an approximately even distribution of reads along the full-sized TE body not emanating from any single TE fragment. Green, yellow, blue and grey lines indicate reads per million (RPM) levels in the control, Piwi-GKD, HP1a-GKD and double-GKD, respectively.

The analysis of the RNA-seq data showed that Piwi-GKD expectedly caused significant derepression of a large set of TEs. Surprisingly, the effect of HP1a-GKD on the TEs was more modest. Of the 125 TE families examined, 22 families displayed a more than two-fold upregulation in the Piwi-GKD, only 10 in the HP1a-GKD and 21 families in the double-GKD ovaries (Table S1). Interestingly, the sets of TEs derepressed upon the three GKD overlapped only partly (Figure 2A–E). The regulation of selected TEs was confirmed by RT-qPCR (Figure S1).

### 2.2. Piwi and HP1a Knockdowns Display Positive Epistatic Interactions for Some TEs 

We divided the TEs according to their responses to Piwi-, HP1a- and double-GKD into four groups (Figure 2A). TEs upregulated more than two-fold upon both Piwi- and HP1a-GKDs were classified as group I (marked in red in Figure 2B–D). This group included mostly LTR retrotransposons (*copia*, *mdg3*, *transpac* and others) and DNA transposon *bari1* (Figure 2E). The RNA-seq profile for a representative of this group *copia* is shown in Figure 2F. As a rule, the elements of this group did not show additional derepression upon double-GKD compared to single GKDs (Figure 2C–E). These observations point to the participation of Piwi and HP1a in a single pathway. Presumably, group I TEs are regulated by canonical piRNA silencing, in which Piwi can initiate the establishment of the H3K9me3 mark with the subsequent binding of HP1a required for transcriptional repression. It is also conceivable that the putative function of HP1a in piRNA biogenesis [48] may contribute to the lack of silencing of these TEs in HP1a- and double-GKD ovaries.

We found as many as 15 TE families, which were more than two-fold upregulated upon Piwi-GKD but not upon HP1a-GKD (group II, marked in dark yellow in Figure 2B–D). The most prominent representative of this group was the LTR element *burdock*, which was more than 20-fold derepressed in Piwi-GKD ovaries and showed no significant changes of expression upon HP1a loss (Figure 2F). Similarly, the TEs of this group, including *burdock* and *blood*, have previously been shown to be upregulated upon germline Piwi depletion [36] and not upon HP1a-GKD [48], whereas another report revealed a moderate derepression of *burdock* and *blood* in HP1a-depleted germ cells [52]. Surprisingly, upon double-GKD, most of group II TEs, albeit not all of them, were expressed at drastically lower levels than in Piwi-GKD ovaries (Figure 2C,E) (for example, see the RNA-seq profile for the *burdock* element (Figure 2F)). Thus, they demonstrated regulation that can be categorised as a positive epistatic interaction between Piwi- and HP1a-GKD. The lack of TE activation upon double-GKD appears not to be caused by a lower knockdown efficiency. According to RNA-seq, the level of *piwi* mRNA was decreased 2.26-fold and 1.94-fold upon Piwi-GKD and double-GKD relative to the Ctrl, respectively, and *HP1a (Su(var)205)* mRNA was reduced 1.84-fold and 1.96-fold upon HP1a-GKD and double-GKD, respectively (Table S2) (the remaining Piwi and HP1a mRNA can be derived from somatic cells of ovaries). The observed epistasis is also unlikely to be attributed to morphological differences between Piwi-GKD and HP1a-GKD/double-GKD ovaries, since we purposefully dissected phenotypically similar ovaries for RNA-seq. Additionally, the influence of morphological discrepancies was excluded by RT-qPCR showing that the upregulation of *burdock* did not differ markedly between Piwi-GKD mature ovaries and 0- to 1-hour-old ovaries containing only germaria and egg chambers of the early stages (data not shown). 

A possible explanation of the observed epistatic interactions could be that HP1a-GKD leads to the downregulation of some transcription factor(s) required for the expression of group II TEs. Therefore, we checked the factors promoting TE transcription among genes that were significantly downregulated upon HP1a-GKD and double-GKD (Table S2). To our knowledge, the putative TE-activating factors in *Drosophila* include caudal (*cad*), dorsal/NF-κB (*dl*), Hsf, tango (*tgo*) [53], Brahma (*brm*) [39,54], PAF1 (*atms*) and RTF1 (*Rtf1*) [55]. Of note, it cannot be excluded that HP1a-GKD may also influence the expression of some uncharacterised TE activators. We revealed that HP1a-GKD and double-GKD led to a 20-fold and 12-fold downregulation of *cad*, respectively, whereas the expression of the other known factors was unchanged (Table S2). Piwi-GKD did not affect the expression of *cad* and the other factors. Cad was shown to be associated with some LTR elements constituting group II in our analysis, including *burdock*, *blood* and *HMS-Beagle*, both by ChIP and bioinformatic predictions [53]. However, some elements belonging to other groups, such as *copia*, also contain binding sites for Cad [53]. Further studies are required to elucidate the role of Cad in TE activation.

Thus, our results suggest that HP1a can be indirectly required for the transcriptional activation of some germline-expressed TEs. This effect can substantially reduce the number of TE families that exhibit upregulation in HP1a-GKD and double-GKD ovaries. 

### 2.3. Piwi-Independent Silencing by HP1a Contributes to Regulation of TEs in Ovarian Germ Cells

Group III consists of TEs that were derepressed >two-fold only upon double-GKD but not upon Piwi-GKD or HP1a-GKD (marked in purple in Figure 2B–D) and includes LTR elements (*frogger*, *aurora*, *gypsy7* and others), as well as the LINE retrotransposon *G-element* (Figure 2E). This type of regulation implies that the repression of these TEs is redundantly controlled by Piwi and HP1a. Hypothetically, upon HP1a loss, these elements can be silenced by H3K9me3/HP1a-independent mechanisms of Piwi-mediated repression, whereas, in Piwi-GKD ovaries, they can be repressed by Piwi-independent HP1a deposition. 

Finally, we revealed three LTR TEs that showed >two-fold upregulation upon HP1a-GKD being significantly less affected by Piwi-GKD: *297*, *1731* and *412* (marked in blue in Figure 2B–D). These TEs were designated as group IV. It should be noted that insertions of *297* and *412* are highly abundant: in the euchromatin of the analysed genome, we found 51 and 46 insertions of the *297* and *412* elements, respectively (Table S3). Interestingly, the highest transcription levels of the *297* and *412* TEs were previously observed in somatic tissues [24,56,57]. The *297* element was shown to be especially active in embryos where it is regulated by Piwi-induced H3K9me3 deposition [24]. Insertions of *297* are also associated with H3K9me3 in ovaries, albeit to a lesser extent compared to embryos [24]. These data suggest that these TEs can be targeted by piRNAs produced specifically in somatic tissues, where they are capable of the strongest transcription. Nevertheless, by normalising RNA-seq to DNA-seq RPMs for different TE families, we found that, in the control (non-GKD) ovaries, the group IV elements were expressed more actively than most elements corresponding to other groups (Table S4). Thus, even if normally transcripts of group IV TEs originate mostly from somatic ovarian cells, their two-fold upregulation upon HP1a-GKD indicates an important role of HP1a in their regulation in germ cells. 

Of note, we did not find a correlation between belonging to a particular group and the number of insertions of a given TE or the ratio of euchromatic insertions against the total number of genomic insertions (data not shown). Moreover, the propensity of a TE family for a particular type of regulation can likely be influenced by the genetic background. For instance, a drastic upregulation of *HeT-A* and other telomeric TEs detected upon HP1a-GKD in Reference [48] was not observed in our analysis, while some TEs (for example, *copia* and *mdg3*) were similarly upregulated. This may be due to differences in the compositions of telomeric chromosome regions between the studied *Drosophila* lines.

Altogether, our results suggest that, along with Piwi-mediated HP1a deposition, some other mechanisms of HP1a-based repression of TEs can operate in the ovarian germline. 

### 2.4. Effects of Piwi and HP1a on Transcription of Euchromatic Loci Flanking TE Insertions 

**Figure 3:**
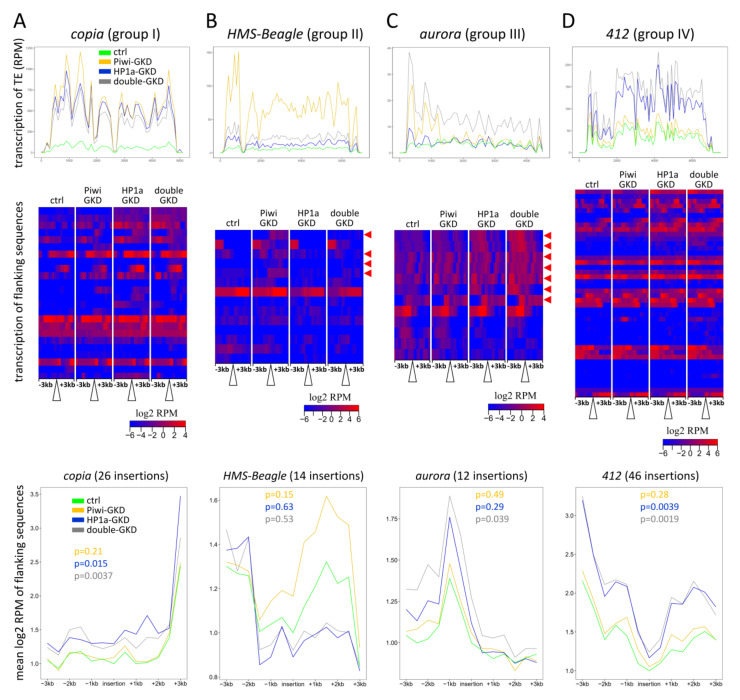
Effects of Piwi and HP1a germline knockdowns on the transcription of genomic loci flanking the insertions of (**A**) *copia*, (**B**) *HMS-Beagle*, (**C**) *aurora* and (**D**) *412* TEs. Upper panels: density profiles of RNA-seq RPM on the TEs in 100-bp windows. Green, yellow, blue and grey lines indicate transcript levels in the control, Piwi-GKD, HP1a-GKD and double-GKD, respectively. Middle panels: heat maps centred at the TE insertion sites showing the transcript levels (log2 RPM) of +/− 3-kb genomic regions flanking the insertions of *copia*, *HMS-Beagle*, *aurora* and *412* in 300-bp windows. Red arrowheads mark *HMS-Beagle* insertions with the most pronounced epistatic upregulation upon Piwi-GKD and *aurora* insertions with the most pronounced upregulation upon double-GKD. Bottom panels: metaplots representing the average RNA-seq signal distribution (log2 RPM) in the flanking regions around all insertions of *copia*, *HMS-Beagle*, *aurora* and *412*. All the values are normalised to the RPM value in the central bin in the control. Green lines indicate the levels in the control, and yellow, blue and grey lines indicate the levels in Piwi-GKD, HP1a-GKD and double-GKD, respectively. The Mann–Whitney *U* test was used for comparison of the average RNA-seq signal in the flanking regions upon Piwi-GKD (yellow), HP1a-GKD (blue) and double-GKD (grey) versus the control.

TE sequences can affect the nearby host genes due to the spreading of repressive chromatin up to 20 kb from the insertions [15,18,19,20,37]. Since the piRNA pathway induces chromatin-based transcriptional silencing of TEs, it can also influence the transcription of genes in the vicinity of TE insertions as was shown in ovarian somatic cells (OSC) [20], whereas only a few examples of such regulation have been described in germ cells [58]. We therefore examined how the studied germline knockdowns affected the transcription of genomic regions adjacent to euchromatic TE insertions. Using PoPoolationTE2 software [59], we identified 4084 TE insertion sites in the analysed genome, 2474 of which were located in euchromatin (Table S3). We then visualised the levels of transcripts (log_2_ RPM) derived from +/− 3-kb genomic regions flanking individual TE copies in euchromatin as heat maps centred at TE insertion sites (Figure 3, middle panels) and as metaplots representing the average RNA-seq signal distribution in the flanking regions around all the insertions of a particular TE family (Figure 3, bottom panels). The flanking regions of many TEs displayed elevated RPM levels that can reflect both the extension of TE-derived transcription or increased expression of genes adjacent to insertions. Different insertions of the same TE exerted highly heterogenous effects, and for many insertions, the flanking regions were not upregulated (Figure 3, middle panels). This heterogeneity may be determined by the presence of active nearby genes and possibly by the chromatin context. We found that the transcriptional spreading from insertions on the whole reflects but does not completely recapitulate the effects on TEs themselves (compare the upper panels with TE profiles with the middle and bottom panels in Figure 3). For example, *copia* belonging to group I TEs was similarly activated upon all the three knockdowns (Figure 3A, upper panel), and accordingly, the transcription of genomic regions adjacent to some *copia* insertions was affected by all GKDs. However, increased spreading from several other *copia* insertions was observed only upon HP1a- and double-GKD but not upon Piwi-GKD (Figure 3A, middle panel). The mean changes of RPM on *copia*-adjacent sequences were statistically significant only for HP1a-GKD and double-GKD (Mann–Whitney test, *p* = 0.015 and *p* = 0.0037, respectively) (Figure 3A, bottom panel). Compared to *copia*, insertions of *mdg3*, another member of group I, showed a stronger effect upon Piwi-GKD, in accordance with the regulation of this TE (Figure S2). The epistatic effects typical for the group II elements can be seen for some *HMS-Beagle* insertions (Figure 3B, middle panel, red arrows). Surprisingly, the flanking regions of several other group II TEs were also upregulated upon HP1a-GKD and double-GKD (for example, some insertions of *3S18* and *burdock*), despite the pronounced Piwi/HP1a epistasis in the regulation of these TEs (Figure S2). These observations suggest that the loss of HP1a can abolish the spreading of repressive chromatin from a TE insertion, even if not accompanied by an increase in TE transcription.

Among the group III TEs, the element most widely represented in euchromatin was *aurora* (12 insertions). Consistent with the regulation of this TE (Figure 3C, upper panel), the transcription of the flanking regions for seven *aurora* insertions was higher upon double-GKD than upon single knockdowns (Figure 3C, middle panel). Accordingly, the averaged effect of upregulation at the flanking regions over all *aurora* insertions was significant only for double-GKD (Mann–Whitney test, *p* = 0.039) (Figure 3C, bottom panel). Piwi-independent regulation, characteristic for group IV TEs, can be exemplified by insertions of the *412* element, whereas the transcriptional spreading upon Piwi-GKD was also observed for several *412* insertions (Figure 3D). 

**Figure 4:**
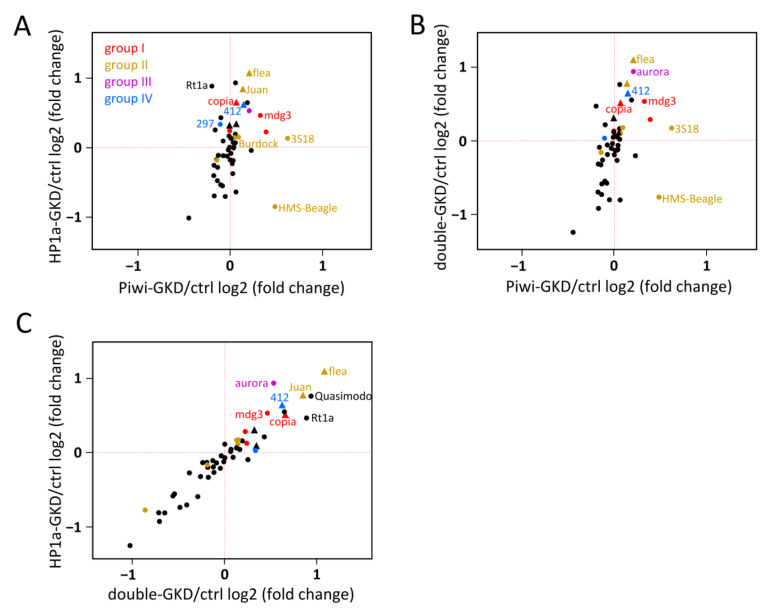
Summarised changes of the transcript levels in genomic loci flanking insertions of different TEs upon Piwi and HP1a germline depletion. (**A**) Scatterplot showing the fold change of the total RNA-seq signals derived from genomic regions flanking (+/− 3 kb) all insertions of each TE family (log2 sum of RPM) upon Piwi-GKD relative to the control versus the fold change upon HP1a-GKD. The top 50 most abundant TEs in euchromatin of the analysed genome are shown. The TEs of group I are marked in red, group II in dark yellow, group III in purple and group IV in blue. *p*-values <0.05 for the mean RPM changes of the flanking regions upon HP1a-GKD are denoted by triangles. (**B**) The same for Piwi-GKD versus Piwi/HP1a double-GKD. (**C**) The same for double-GKD versus HP1a-GKD.

To compare the spreading effects for different TEs, we calculated total changes in the level of transcripts from genomic regions flanking (+/− 3 kb) all insertions of each TE family (sum of RPM in GKD relative to the control ovaries) (Figure 4). To minimise the influence of gene expression, which may be changed due to indirect knockdown effects, we selected the top 50 most abundant TEs in the euchromatin of the analysed genome. For each of these TEs, at least 12 insertions were found. Although the mean RPM changes of the flanking regions were not significant upon Piwi-GKD, which is apparently due to the fact that only a small fraction of insertions affect transcription in the nearby genome, the results in Figure 4A imply that Piwi exerts a repressive effect on the genomic regions near Piwi-regulated TEs. In Piwi-GKD ovaries, the transcription of adjacent genomic sequences for nine out of 11 TEs corresponding to groups I and II displayed a slight increase relative to the Ctrl (Figure 4A), which was not observed for many TEs that were not activated upon the knockdowns (black dots in Figure 4A). The regions flanking most TEs of groups I and II, with a notable exception of *HMS-Beagle*, were also upregulated upon HP1a-GKD, suggesting the cooperative role of Piwi and HP1a in the spreading of repressive chromatin from TE insertions (Figure 4A–C). Notably, HP1a-GKD affected the transcriptional spreading of some Piwi-regulated TEs stronger than Piwi-GKD (*p*-values < 0.05 for the mean changes of RPM upon HP1a-GKD are denoted by triangles in Figure 4). Interestingly, the averaged RNA-seq signal around the insertions of all the TE families was increased significantly only in the HP1a-GKD and double-GKD ovaries (Mann–Whitney test, *p* = 0.00037 and *p* = 0.0014, respectively) but not upon Piwi loss (*p* = 0.97) (Figure S3). 

Collectively, these results suggest that both Piwi and HP1a are required for the repression of genomic regions adjacent to TE insertions in germ cells, while HP1a can play a more substantial role in this process.

### 2.5. Contribution of Piwi and HP1a-Mediated Repression of Transposons to the Regulation of Protein-Coding Genes in the Germline

**Figure 5:**
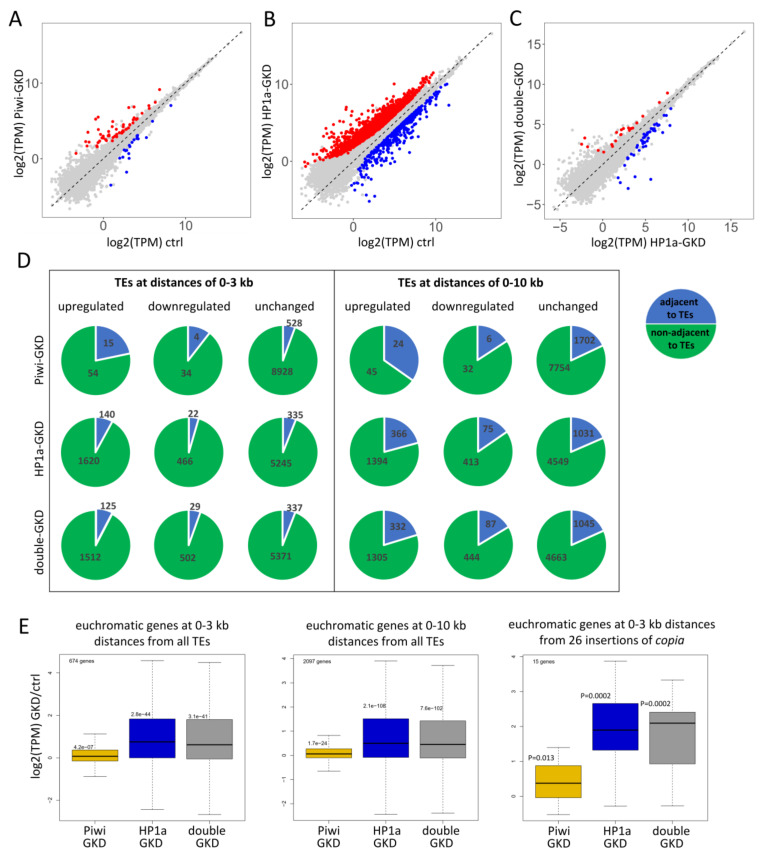
Contribution of Piwi and HP1a to the regulation of protein-coding genes in the germline. (**A**,**B**) Scatterplots showing transcript levels (log2 TPM) of genes upon Piwi-GKD (**A**) and HP1a-GKD (**B**) versus the control. Significantly up- and downregulated genes are indicated by red and blue dots, respectively. (**C**) Scatterplot showing the expression of genes in HP1a-GKD versus Piwi/HP1a double-GKD ovaries. (**D**) Number of euchromatic genes that were >2-fold upregulated, downregulated or did not change expression upon Piwi-, HP1a- and double-GKD. Blue sectors show the number of genes adjacent to TE insertions at distances up to 3 kb or 10 kb, and green sectors show the number of genes nonadjacent to TE insertions. (**E**) Boxplots indicating fold changes (log2) in the RNA-seq TPM values upon Piwi-, HP1a- and double-GKD for euchromatic genes adjacent to the insertions of all TEs at distances up to 3 kb or 10 kb and for euchromatic genes adjacent to insertions of the *copia* element at a distance of up to 3 kb. In the boxplots, the median is represented by a thick horizontal line, the upper and lower hinges are the 75th and 25th percentiles, respectively, and the whiskers indicate the highest and lowest values. *p*-values were calculated according to the Mann–Whitney U test.

We investigated the extent to which Piwi and HP1a-dependent chromatin spreading from TEs can affect the expression of host genes. DESeq2 analysis revealed that 103 protein-coding genes were significantly upregulated and 67 downregulated upon Piwi-GKD (Figure 5A and Tables S2 and S5). HP1a-GKD affected the expression of a large number of genes: 2184 were upregulated and 2174 downregulated according to the adjusted DESeq2 *p*-values (Figure 5B and Tables S2 and S5). Among them, the expression of 1760 and 488 genes showed a >two-fold increase and decrease, respectively. Double-GKD influenced gene expression similarly to HP1a-GKD (Figure 5C and Tables S2 and S5). These results are in accord with the previously observed huge changes in gene expression due to the loss of HP1a both in the ovarian germline [50] and in other *Drosophila* tissues and cultured cells [14,60,61,62]. 

We divided the protein-coding genes into euchromatic and heterochromatic, the latter genes localised in constitutive heterochromatin at pericentromeric regions of the chromosomes. According to the DESeq2 adjusted p-values, among the 245 heterochromatic genes with detectable expression in the analysed transcriptomes, 58 genes were significantly upregulated, and 50 genes were downregulated in HP1a-GKD ovaries. However, among the genes that showed >two-fold change in expression, 47 were upregulated and only seven were downregulated (Table S5). Consistent with previous reports [60,63], the loss of HP1a also caused a strong upregulation of genes localised in the predominantly heterochromatic small chromosome 4: 26 of the 73 genes were activated, and none were downregulated more than two-fold (Table S5). Piwi-GKD affected the expression of only eight heterochromatic genes and did not influence the genes of chromosome 4 at all (Table S5).

Next, we evaluated whether the differentially expressed euchromatic genes are located close to insertions of TEs. We found that most upregulated and downregulated euchromatic genes upon all the three knockdowns, and especially upon HP1a-GKD and double-GKD, are not adjacent to TEs at least at a distance of up to 10 kb (Figure 5D and Table S5). These results indicate that the observed changes in gene expression upon HP1a loss are mostly not associated with the regulation of TEs. The molecular mechanisms by which HP1a and other components of heterochromatin can affect many euchromatic genes independently of TEs are discussed in a number of works [14,50,51,58,64] but generally remain unclear (see Section 3). Nevertheless, we suppose that a minority of genes can be upregulated due to the abolishment of repressive chromatin spreading from TE insertions. This is supported by the fact that the proportion of genes residing near TE insertions is noticeably higher for the upregulated genes than for the downregulated ones and genes with unchanged expression (Figure 5D and Table S5). This effect was observed upon all three knockdowns but was most pronounced for Piwi-GKD.

We then investigated the influence of individual TE families on gene expression upon the tested knockdowns. We considered only TE families with euchromatic insertions adjacent to at least 10 genes at distances up to 3 or 10 kb. For 15 and 41 TE families, we detected a significant elevation of expression of 0–3- or 0–10-kb adjacent genes upon HP1a-GKD, respectively (*p* < 0.05, Mann–Whitney test for expression increase upon knockdown compared to the control) (Table S). In the case of Piwi-GKD, there were fewer such TE families (8 and 16 for distances up to 3 or 10 kb, respectively), and the genes were upregulated weaker than upon HP1a-GKD and double-GKD (Figure 5E and Table S6). However, the expression of genes adjacent (0–10 kb) to Piwi-sensitive TEs, including *burdock*, *3S18*, *juan*, *HMS-Beagle*, *jockey* and *transpac*, was significantly affected upon both Piwi- and HP1a-GKD (Table S6), confirming the cooperative role of Piwi and HP1a in repressive spreading from TE insertions. To check whether the activation of these genes is indeed attributed to their proximity to TE insertions, we took the same number of random genes in the genome as the number residing nearby each TE family and calculated the *p*-value for their upregulation, repeating this action for 10,000 random gene subsets. The resulting *p*-value in this test was the number of times random genes showed more significant upregulation than the actual gene set divided by 10,000 (Table S7). This test showed that the observed effects of activation of the genes adjacent to most TE families upon HP1a-GKD may not be associated with TE insertions (*p*-value > 0.05). However, the probability of upregulation of the genes adjacent to insertions of the *copia* element at a distance up to 3 kb was significantly higher than random upon both HP1a-GKD and double-GKD (*p*-value < 0.05) (Table S7). The effects of Piwi-GKD for genes residing at a distance of up to 10 kb from TE insertions appeared to be non-random for three Piwi-regulated TEs belonging to group II: *burdock*, *3S18* and *juan* (Table S7). Surprisingly, Piwi-GKD also non-randomly affected the genes residing close to the insertions of the *412* elements, despite the transcript level of this TE belonging to group IV increasing by only 12% upon Piwi-GKD (Table S1), and only a few of its insertions displayed elevated transcriptional spreading upon Piwi loss (Figure 3D). Thus, the upregulation of a number of genes upon Piwi-GKD undoubtedly takes place due to the presence of adjacent TE insertions. The fact that we failed to find this dependence for HP1a is likely caused by the global influence of HP1a loss on low expressed genes, which does not allow us to statistically reveal the effect of HP1a on genes near the insertions. However, the strong effects of HP1a-GKD on transcriptional spreading from TEs (Figure 3 and Figure 4) and the higher proportion of upregulated genes among TE-adjacent genes (Figure 5D) suggest that HP1a is involved in gene repression mediated by TE insertions.

### 2.6. Piwi/HP1a-Dependent Silencing of TEs Is Implicated in Tissue-Specific Gene Repression

**Figure 6:**
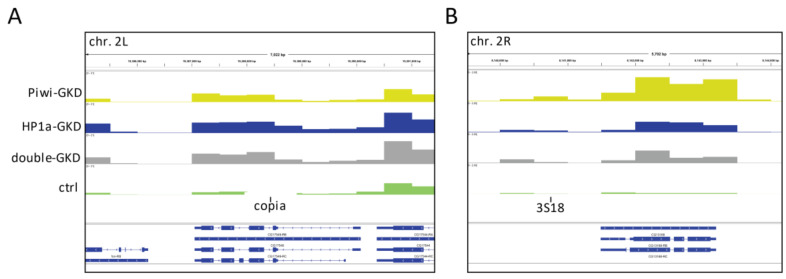
Examples of genes silenced in the germline through Piwi/HP1a-dependent spreading from TE insertions. Screenshots of the genome browser showing RNA-seq signal distribution in 500-bp windows for the CG17549 (**A**) and CG13168 (**B**) genes and neighbouring genomic regions. Insertion sites of *copia* and *3S18* TEs are indicated. Green, yellow, blue and grey tracks show transcript profiles in the Ctrl, Piwi-GKD, HP1a-GKD and double-GKD, respectively.

The observed modest changes in the gene transcript levels upon Piwi-GKD imply that Piwi-dependent gene regulation is unlikely to be widespread in the germline (Figure 5D and Table S5). We found only 14 euchromatic genes that reside near TE insertions at distances up to 10kb and are upregulated >two-fold upon separate knockdowns of both Piwi and HP1a (Table 1 and Table S8). These genes can be influenced by TEs via Piwi-induced chromatin silencing. The insertions were located in both introns of these genes and adjacent intergenic regions (Table 1) and, in half of the cases, corresponded to TEs for which we found non-random effects on gene expression (Table S7). Interestingly, according to modENCODE_mRNA-Seq_tissues [65], most of these genes (12 out of 14) are characterised by the high or very high expression in different organs, for example, head, accessory gland, fat body, digestive system, testes, etc. (Table 1). However, these genes are almost not transcribed in ovarian cells: 12 genes display extremely low/no expression, and two exhibit low expression levels in ovaries [65]. Consistent with these data, our RNA-seq analysis showed that more than half of these 14 genes were expressed at levels less than 1 TPM (transcript per million) in the control ovaries. Their median expression was 0.7 TPM, while the median expression of all genes was 12.5 TPM. In Piwi-GKD ovaries, the median expression of this gene subset increased to 9.3 TPM (with a median expression of all the genes of 13.2 TPM); upon HP1a-GKD, it increased to 5.2 TPM (the median for all genes was 17.6 TPM) and, upon double-GKD, to 10.0 TPM (the median for all genes was 16.5 TPM). Thus, upon the knockdowns, most of these genes moved from extremely low to moderate expression. A screenshot of the genome browser in Figure 6A exemplifies the regulation of the CG17549 gene containing an intronic insertion of *copia*. CG17549 is known to be highly expressed in several structures, including the foregut, accessory gland and nervous system, but has a dramatic drop in expression level in ovaries [66]. Another example is CG13168, which is likely inactivated by repression spreading from the *3S18* element inserted into the intergenic region (Figure 6B). This gene is highly transcribed in imaginal disks, testes and fat body [65]. 

Thus, Piwi-dependent HP1a deposition may regulate the expression of some genes in the germline by the spreading of the repressive influence from TE insertions. Most of these genes are characterised by extremely low transcription in ovaries but show a high level of transcription in other organs, which, at least partially, can be explained by the fact that repression by germline-specific piRNAs is lost in other tissues. These results suggest that Piwi-mediated spreading of repressive chromatin can prevent ectopic transcription of inappropriate genes in ovaries.

## 3. Discussion

Substantial parts of eukaryotic genomes are composed of TE sequences, which are considered as one of the main drivers of evolution due to their ability to induce changes in DNA and to alter the expression of nearby genes [15,16,18,20,67]. In this work, we assess the contribution of epigenetic TE regulation through the Piwi-piRNA pathway and the main heterochromatin protein HP1a to the overall picture of gene regulation in the *Drosophila* germline. 

By evaluating the effects of Piwi and HP1a double or separate knockdowns in similar genetic backgrounds, we revealed that certain TE families tend to be predominantly Piwi- or HP1a-regulated (Figure 2). In the course of piRNA-induced chromatin silencing, Piwi is known to recruit the SFiNX/Pandas/PICTS/PNNP complex, which then deposits H3K9me3/HP1a marks at target loci [44,45,46,47]. In addition, Piwi-silenced TEs in the germline are associated with protein complexes, which prevent normal mRNA synthesis and instead license noncanonical transcription, producing piRNA precursors [38,68,69,70]. Although some TEs in our analysis behaved consistently with the participation of Piwi and HP1a in a single pathway (group I, see the model in Figure S4), a subset of Piwi-dependent TEs was much more weakly activated upon HP1a loss and, more surprisingly, upon Piwi/HP1a double-GKD (group II, Figure 2C). Since HP1a-GKD induces changes in the expression of many protein-coding genes, we hypothesised that it could downregulate some TE-activating transcription factors and suggested Cad as a candidate for this role (Figure S4). Thus, it is possible that the repression of group II TEs occurs via the same Piwi-induced HP1a deposition mechanism, but downregulation of the transcription factors prevents the activation of the canonical transcription of these TEs upon the loss of HP1a. This hypothesis may explain why we and other studies [48,52] detected a rather modest upregulation of TEs upon germline depletion of HP1a. Interestingly, the genomic regions and genes adjacent to some insertions of group II TEs displayed elevated transcription in the HP1a-GKD ovaries (Figure 4 and Figure S2 and Table S6). This may be due to the fact that the loss of HP1a abolishes the spreading of repressive chromatin from TE insertions to nearby genes, even if the TEs themselves are not upregulated (Figure S4).

Previously, many TEs, which are not susceptible to Piwi-mediated silencing, have been found to be enriched with HP1a and derepressed upon its depletion in the somatic cells of ovaries [14,49]. Our results indicate that piRNA-independent targeting of TEs by HP1a can also operate in ovarian germ cells given that some TEs are upregulated upon HP1a- or double-GKD but not upon Piwi loss (Figure S4). Interestingly, the H3K9me3 marks deposited at the *roo* element by the piRNA pathway in *Drosophila* embryos have been shown to not be epigenetically inherited during the subsequent cell divisions, making unlikely the possibility that the repressive chromatin state can be established by Piwi, and then is maintained by HP1a in a Piwi-independent manner [24]. To our knowledge, apart from piRNAs, no other molecular mechanisms by which euchromatic TE insertions in *Drosophila* can be recognised and repressed at the chromatin level have been described so far. The role of the endo-siRNA pathway cannot be ruled out, although the analysis of wild type *Drosophila* strains did not reveal significant associations between the epigenetic effects of TEs and targeting by endo-siRNAs in contrast to piRNAs [15]. In addition, it is tempting to speculate that the recruitment of H3K9me3/HP1a at some TEs can be promoted by DNA-binding proteins capable of recognising specific DNA motifs by analogy with mammalian KRAB-Zinc finger repressors (reviewed in Reference [71]).

Huge alterations of the gene expression have been observed due to the disruption of various heterochromatin components [14,50,58,60,61,62]. Here, we showed that most gene expression changes upon HP1a loss in the germline were not coupled with TEs. HP1a is thought to be implicated in the regulation of euchromatic genes by the stabilisation of their mRNAs [11,51]. Another possibility is that HP1a can be bound to the H3K9me3 marks deposited at euchromatic sites in a TE-independent manner [58]. However, we previously found that most genes changing expression due to HP1a loss in somatic ovarian cells are not directly targeted by HP1a [14]. It is also conceivable that gene regulatory networks can be disrupted upon HP1a loss as a result of the dysregulation of key transcription factors, which, however, leaves open the question of how HP1a regulates these factors themselves. For example, the inappropriate activation of a substantial number of testis-specific genes in ovaries upon the germline knockdown of Egg/SetDB1 (H3K9me3 histone methyltransferase) is a consequence of the ectopic expression of the PHF7 transcriptional regulator [50]. However, we did not observe the upregulation of phf7 upon HP1a-GKD (Table S2).

TEs are known to serve as seeding points for H3K9me2/3 and HP1a deposition in euchromatin [14,15,19,20]. More than half of euchromatic TE insertions have been shown to spread repressive chromatin marks, and at least in some cases, this spreading can influence the expression of adjacent genes [15,18]. In cultured ovarian somatic cells, the Piwi-piRNA pathway regulates several dozens of protein-coding genes by inducing euchromatic H3K9me3 islands at TE sequences [20]. Here, we showed that separate or simultaneous germline knockdowns of Piwi and HP1a increased the transcription levels of genomic sequences flanking insertions corresponding mainly to the Piwi-repressed TEs (Figure 4). We found that a small set of protein-coding genes containing TE insertions within introns or residing near TEs are significantly upregulated upon Piwi-GKD, as well as upon HP1a-GKD. These genes can be silenced by the repressive chromatin deposited through Piwi around the TE insertions. However, it also cannot be ruled out that enhanced TE transcription caused by the loss of Piwi or HP1a can spread to these genes, inducing their abnormal upregulation. Interestingly, these target genes are characterised by high levels of tissue-specific expression but are mostly not transcribed in ovaries (Table 1). Thus, it is plausible that piRNA-dependent spreading of the repressive chromatin state from TE insertions can serve as a mechanism that blocks the transcription of inappropriate genes in ovarian cells. This silencing can be abolished in most other tissues, because they lack Piwi-piRNA complexes or at least those of them that recognise TEs expressed in the ovarian germ cells. Loss of the repressive influence of the piRNA pathway along with the presence of tissue-specific transcription factors can permit the expression of these genes. Further research may elucidate whether this regulation is biologically relevant. In particular, it remains unclear to what extent the ectopic expression of these genes may be deleterious to ovarian germ cells. Nevertheless, this hypothetical phenomenon is interesting from an evolutionary point of view. The piRNA-silencing pathway that operates predominantly in gonadal cells due to the tendency of TEs to transpose in the precursors of gametes may be adapted to play the role of a gonad-specific repressor of host genes.

## 4. Materials and Methods

### 4.1. Fly Stocks

*Drosophila melanogaster* stocks were maintained on the medium containing semolina, sugar, raisins, yeast and agar supplemented with nipagin, propionic acid, streptomycin and benzylpenicillin. The following UAS-RNAi lines received from the Vienna Drosophila Resource Center (VDRC, Vienna, Austria) were used: UAS-Piwi-RNAi (#101658 VDRC) and UAS-HP1a-RNAi (#31994 VDRC). To produce the analysed individuals, females of the previously obtained line with the genotype *UAS-Piwi-RNAi/CyO; UAS-HP1a-RNAi/TM3,Sb1 Ser1* were crossed with males of the *nos-GAL4* stock *P{UAS-Dcr-2.D}1*, *w^1118^*, *P{GAL4-nos.NGT}40* (#25751 Bloomington Drosophila Stock Center, Bloomington, IN, USA), providing germline-specific GAL4 expression under the control of the *nanos* (*nos*) gene promoter together with Dcr2. Crosses were performed at 18 °C, since maintaining them at 25 °C resulted in an almost complete loss of the germ cells in HP1KD and double-KD ovaries. All analysed females (Ctrl, Piwi-GKD, HP1a-GKD and double-GKD) were obtained simultaneously from the same cross.

### 4.2. Immunostaining

Immunostaining of ovaries was performed as previously described [72]. As primary antibodies, rabbit anti-HP1a polyclonal antibodies (PRB-291C, Covance, USA, 1:500), rat anti-Vasa monoclonal antibodies (1:150, DSHB Hybridoma Bank, Iowa City, IA, USA) and mouse monoclonal anti-Piwi antibodies P3G11 (1:500, a gift from M. Siomi [73]) were used. As secondary antibodies, Alexa Fluor 633-conjugated goat anti-rabbit (1:500, Invitrogen, USA), Alexa Fluor 546–conjugated goat anti-rat (1:500, Invitrogen, USA) and anti-mouse IgG Alexa Fluor 488 (1:500, Invitrogen, USA) antibodies were used. Confocal microscopy was performed using the LSM 510 META system (Zeiss, Germany).

### 4.3. RT-qPCR

Total RNA was isolated from manually dissected ovaries using Trizol reagent (Invitrogen, USA) and cleared of genomic DNA by the DNA-free kit (Ambion, USA). One microgram of total RNA was used in the reverse transcription reaction with random hexamer primers by Superscript II reverse transcriptase (Invitrogen, USA). cDNA was analysed on the DT96 real-time DNA amplifier (DNA-Technology, Russia) using SYTO™ 13 Green Fluorescent Nucleic Acid Stain (Thermo Fisher Scientific, USA). The RT-qPCR values were normalised to those of the rp49 mRNA. The following primers were used for PCR: mdg3_s1 GACCGTTCAAAAGTATCTCC and mdg3_as1 TCCTGACAACTAGATCTCCC:; Burdock_fw CGGTAAAATCGCTTCATGGT and Burdock_rw ACGTTGCATTTCCCTGTTTC; Frogger s2 CACCACGAAGACGAAGCAGCA and Frogger as2 TTGACCAGCTCGCCGGTCTT; 1731_fw AGCAAACGTCTGTTGGAAGG and 1731_rv CGACAGCAAAACAACACTGC and Rp49_up ATGACCATCCGCCCAGCATAC and Rp49_rev2 GCTTAGCATATCGATCCGACTGG.

### 4.4. Sequencing and Bioinformatics Analysis of Drosophila Genome

To detect TE insertion sites in the line with germline knockdowns of HP1a and Piwi, we sequenced the genome of double-GKD females with the genotype *P{w[+mC]=UAS-Dcr-2.D}1, w [1118]/+; UAS-Piwi-RNAi(#101658 VDRC)/P{w[+mC]=GAL4-nos.NGT}40; UAS-HP1a-RNAi(#31994, VDRC)/+*. The identified TE insertions sites in this genome were then used for a transcriptomic analysis of the four studied genotypes (Ctrl, Piwi-GKD, HP1a-GKD and double-GKD). 

To perform whole-genome sequencing, genomic DNA was isolated from 25 whole female flies according to the standard procedure [74]. The paired-end library of the fragmented genomic DNA was prepared according to the Illumina standard protocol and sequenced on the Illumina HiSeq 4000 platform on the basis of the Genomics Core Facility of Skolkovo Institute of Science and Technology (Moscow, Russia). Sequencing data were deposited to NCBI Gene Expression Omnibus (GEO) under the accession number GSE186867. In total, ~8.5 million of 101-nt paired-end reads were obtained. The insertion sites of TEs were identified by aligning the reads with the consensus sequences of TEs (http://www.fruitfly.org/p_disrupt/TE.html, accessed on 13 December 2021) and the annotated TE insertion sites in the FlyBase (r.5.57) reference genome using the pipeline PoPoolationTE2 [59]. Overall, 4084 TE insertion sites, 2474 of which are located in euchromatin, were identified (Table S3). 

### 4.5. RNA-seq Procedure and Bioinformatics

For RNA isolation, approximately 20–50 ovaries from 0- to 1-day-old Ctrl, Piwi-GKD, HP1a-GKD and double-GKD females were manually dissected in cold PBS. RNA was isolated using Trizol reagent (Invitrogen, USA), yielding over 2 µg for each genotype, in two biological replicates. rRNA was depleted using the Illumina Ribo-Zero rRNA Removal Kit (Epicentre, Madison, WI, USA). The cDNA library was prepared with random primers, and single-end deep sequencing was carried out on the Illumina HiSeq 2000 in the Laboratory of Evolutionary Genomics, Faculty of Bioengineering and Bioinformatics, Lomonosov Moscow State University (Moscow, Russia). 15.1–18.5 million reads (after leaving out the rRNA reads) were obtained per library. The 50-nt reads were aligned to dm3/R5 genome and transcriptome assembly via STAR [75], counted for the genes of the BDGP5.78 annotation and converted to transcripts per million (TPM) values using Salmon [76] or mapped to TE consensus sequences using HISAT2 [77]. Differential expression was performed by the DESeq2 [78] package for R with the cut-off parameters *p* < 0.05 and, in some cases, TPM fold change >2. Heterochromatic genes were defined as genes residing on chromosomes 4, U, U extra, chr2RHet, chr2LHet, chr3RHet, chr3LHet and chrXHet, as well as residing in heterochromatin on chromosome arms 2R, 2L, 3R, 3L and X, according to the cytological borders of heterochromatin listed in Reference [79]. RNA-seq data were deposited in NCBI GEO under the accession number GSE186867.

### 4.6. Data Visualisation

We visualised the transcript levels (log2 RPM) derived from the +/− 3-kb genomic regions flanking individual TE copies as heat maps centred at the TE insertion sites. For this, we used the genomation package for R [80]. For each of the treatments, we replaced the zeroes with the minimal RPM value found across all the genotypes to be able to use logarithmic transformation.

To prepare the metaplots centred at the TE insertions, normalised RPM values around the TE insertions were obtained as follows. The genome was split into 500-nt bins, RPM values for the control and three knockdowns in the bin containing the insertion (central bin), as well as 6 adjacent bins in each direction, were taken and lined up according to the insertion orientation (+ or − strand). In order to compare transcription activation with the control, we normalised all the values onto the RPM value in the central bin in the control. If that value was below 1, we added 1 to every RPM value to avoid giving too much credit to TEs with low RPM values around them. Then, all the resulting values were averaged for every TE family or for all the TE insertions.

Boxplots with ratios of log2 TPM values of genes residing either in the neighbouring 3 kb or 10 kb of a TE insertion in knockdowns relative to the control were plotted for each TE family that had at least 10 genes in the vicinity of its insertions.

For visualisation of the sequencing tracks, the IGV genome browser was used.

### 4.7. Statistical Analysis

The Mann–Whitney (M–W) *U* test was used either for comparisons of two sample distributions or for comparing the median of distributions with zero. The RPM on the TE-adjacent sequences were averaged over all the insertions and compared between the knockdowns and control. For the analysis of the upregulation of genes adjacent to the TEs, we used one-tailed M–W tests for the log2 ratios of the TPM values upon knockdowns to the control compared with zero as the median. To assess whether the activation of genes residing near TE insertions is indeed attributed to such localisation, we used the permutation (non-random) test. First, we calculated the *p*-value of the activation of genes that were near TEs via the M–W *U* test of their ratio of log2 TPM values in the knockdowns to log2 TPM values in the control for each TE family. Then, we took the same number of random genes and assessed their activation in a similar manner 10,000 times. The resulting *p*-value was the number of times the *p*-value of random gene activation was less than the *p*-value of actual gene activation divided by 10,000.

## Figures and Tables

**Table 1:** List of genes adjacent to TE insertions (up to 10kb) and upregulated >2-fold upon Piwi and HP1a GKD.

Gene Name	Transcript Level (TPM)				Adjacent TE (0–10 kb)	TE Location	Maximum Expression (According to flybase.org)
	Ctrl	Piwi-GKD	HP1a-GKD	Double-GKD			
CG43391	0	44.43	5.13	28.72	*juan*, *jockey*	intergenic region	embryo 22–24 h, head
CG17549	9.31	22.86	40.98	36.45	*copia*	intron	accessory gland
CG32814	5.35	18.56	13.69	13.94	*baggins*, *FB*, *Rt1c*	intron	ovary
CG3348	2.14	5.18	31.93	27.83	*jockey*	intron of neighbouring gene	digestive system
CG34002	0.79	9.29	2.86	7.66	*invader4*	intron, intergenic region	accessory gland
CG34430	0	3.06	0.33	2.00	*mdg3*	intergenic region	N/A
CG42319	0.23	4.80	2.47	2.16	*copia*	intergenic region	carcass, 1-day adult
CG13168	0.25	3.20	1.59	1.40	*3S18*	intergenic region	testis
CG7296	0.65	45.17	2.09	5.63	*412*	intergenic region	carcass, larvae L3
CG9380	3.10	9.40	12.57	12.36	*1360*	intron	digestive system
CG9616	0	1.01	5.25	3.05	*jockey*, *idefix*	intergenic region	fat body, larvae L3
CG9766	0.27	11.29	2.01	2.74	*burdock*	intron	testis
Cyp12a4	2.11	18.93	17.59	30.32	*412*, *bari1*	intergenic region	digestive system
TfIIA-S-2	2.28	6.97	29.04	20.79	*transib4*	intergenic region	testis

## Data Availability

RNA-seq and DNA-seq data were deposited in NCBI GEO under accession number GSE186867.

## Supplementary Materials

The following are available online at https://www.mdpi.com/article/10.3390/ijms222413430/s1.
