# Supplementary material for: Complex Genetic Interactions between Piwi and HP1a in the Repression of Transposable Elements and Tissue-Specific Genes in the Ovarian Germline
Source: Int J Mol Sci. 2021 Dec 14;22(24):13430. doi: 10.3390/ijms222413430 (PMC8707237; doi:10.3390/ijms222413430)
Supplement: Supplementary file 1 [file ijms-22-13430-s001.zip › Saplementary Figures.pdf]

Figure S1

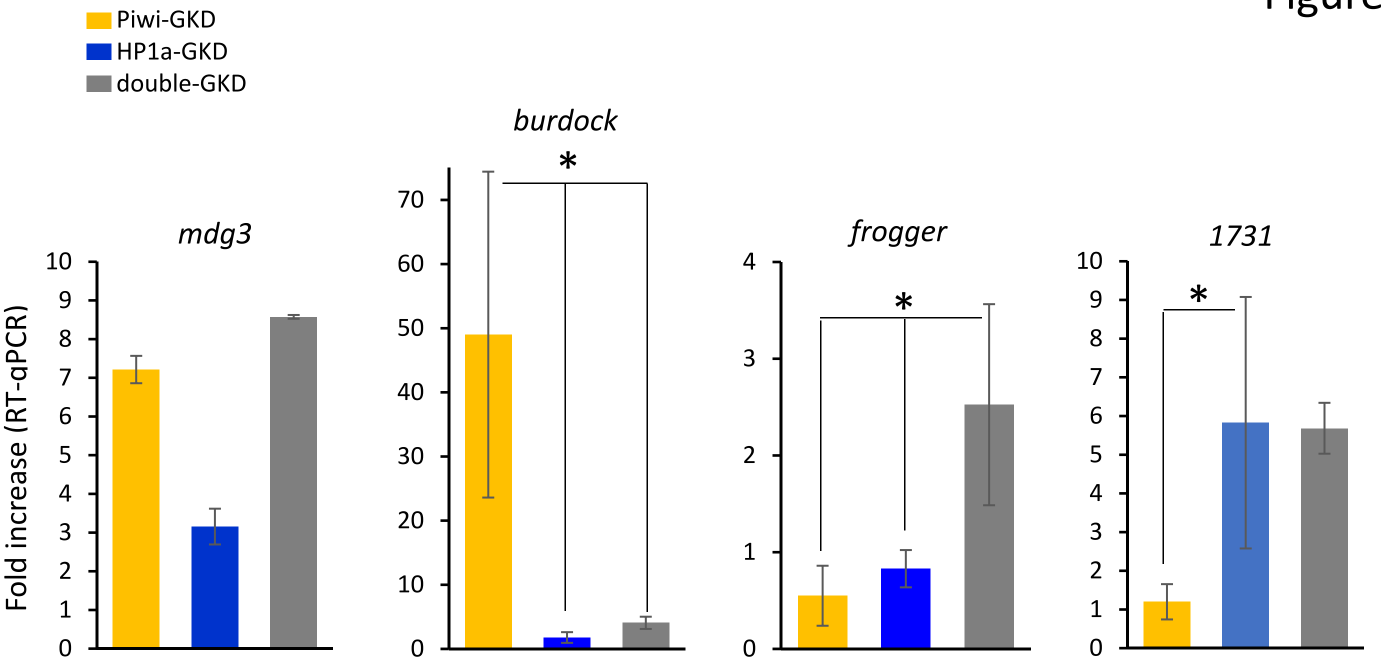

Figure S1. RT-qPCR results showing fold increase of *mdg3, burdock, frogger,* and *1731* transcript levels upon Piwi-GKD (yellow bars). HP1a-GKD (blue bars) and double-GKD (grey bars) relative to control sisters and normalized to rp49 mRNA. Mean +/- s.d. are indicated (Student’s t-test; *p < 0.05).

Figure S2

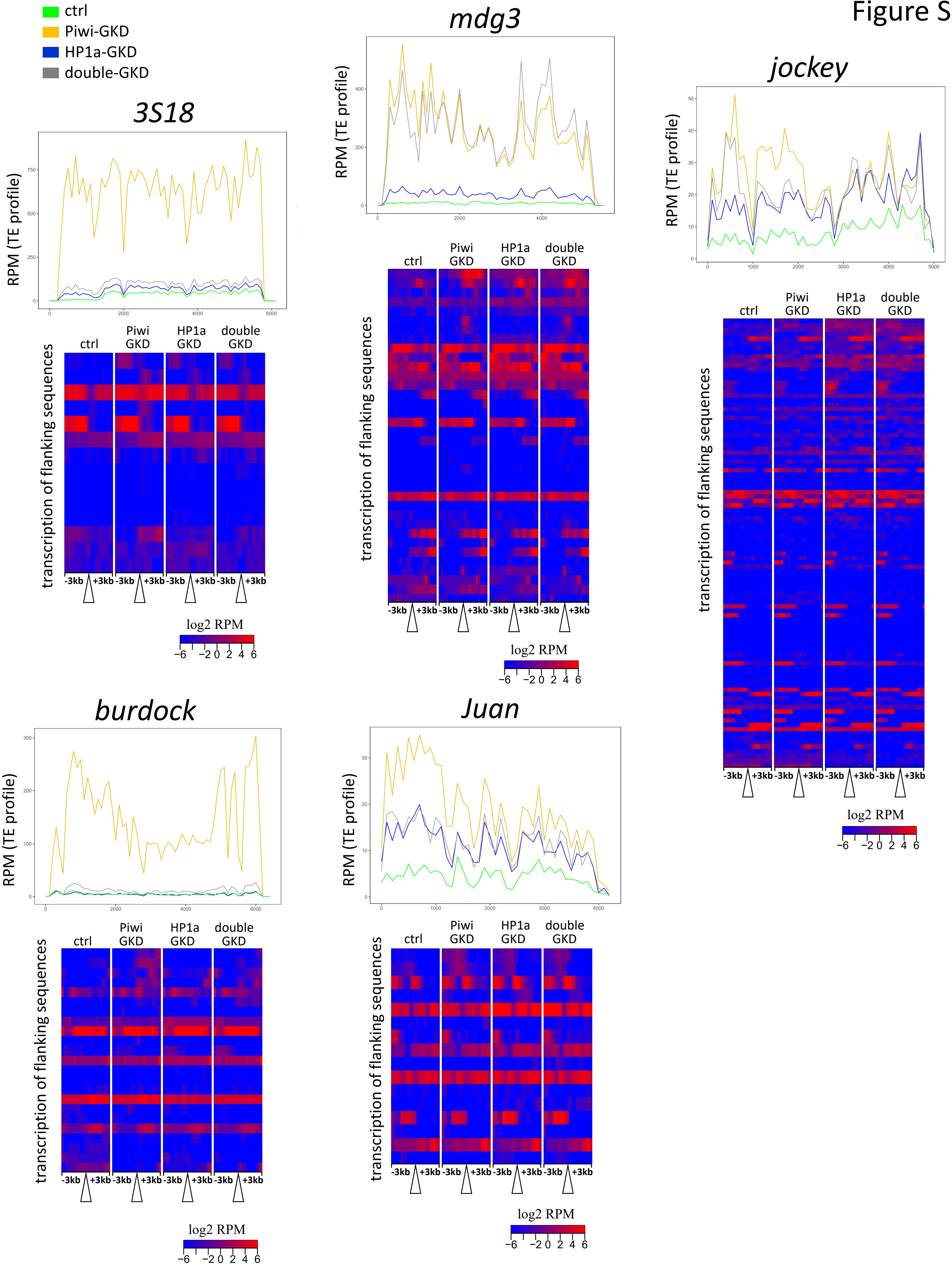

Figure S2. Effects of Piwi-, HP1a-, and double-GKD on expression of *3S18, mdg3, jockey, burdock, and Juan* TEs and genomic loci flanking their insertions. Upper panels: density profiles of RNA-seq RPM on TEs in 100 bp windows. Green, yellow, blue, and grey lines indicate transcript levels in ctrl, Piwi-GKD, HP1a-GKD, and double-GKD, respectively. Bottom panels: heat maps in 300 bp windows centered at TE insertion sites showing transcript levels (log2 RPM) of +/- 3kb genomic regions flanking insertions.

Figure S3

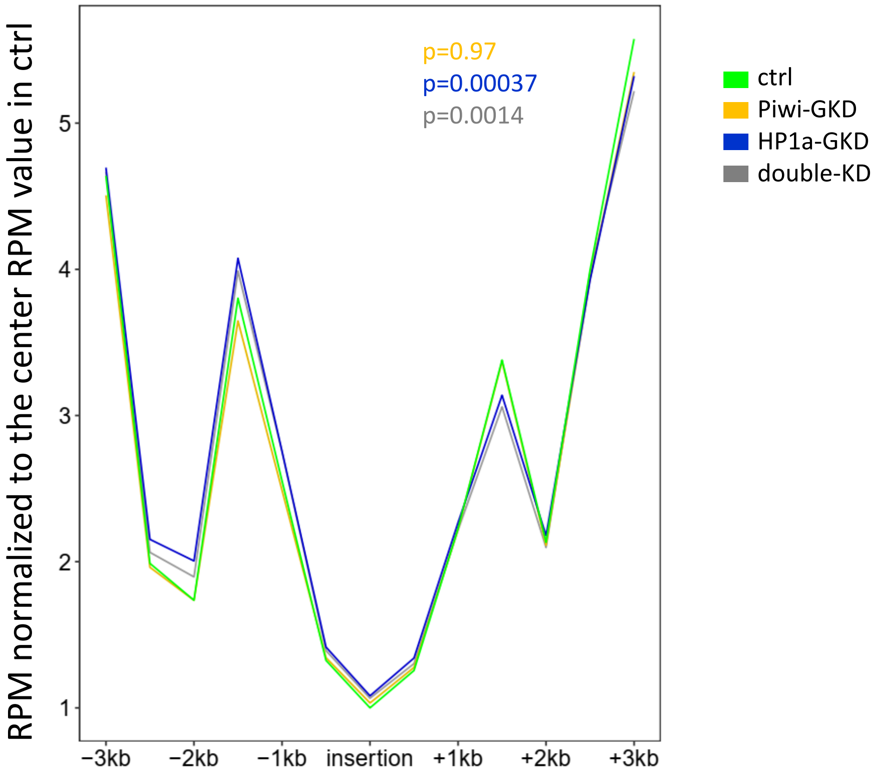

Figure S3. Metaplot representing an overall RNA-seq signal distribution (log2 RPM) around insertions (+/- 3kb) of all TE families. Green, yellow, blue, and grey lines indicate the transcription levels in control, Piwi-GKD, HP1a-GKD, and double-GKD, respectively. The values for each insertion are normalized to the RPM value in the central bin in control. Mann-Whitney U test was used for comparison of the average RNA-seq signal in flanking regions upon Piwi-GKD (yellow), HP1a-GKD (blue), and double-GKD (grey) versus control.

Figure S4

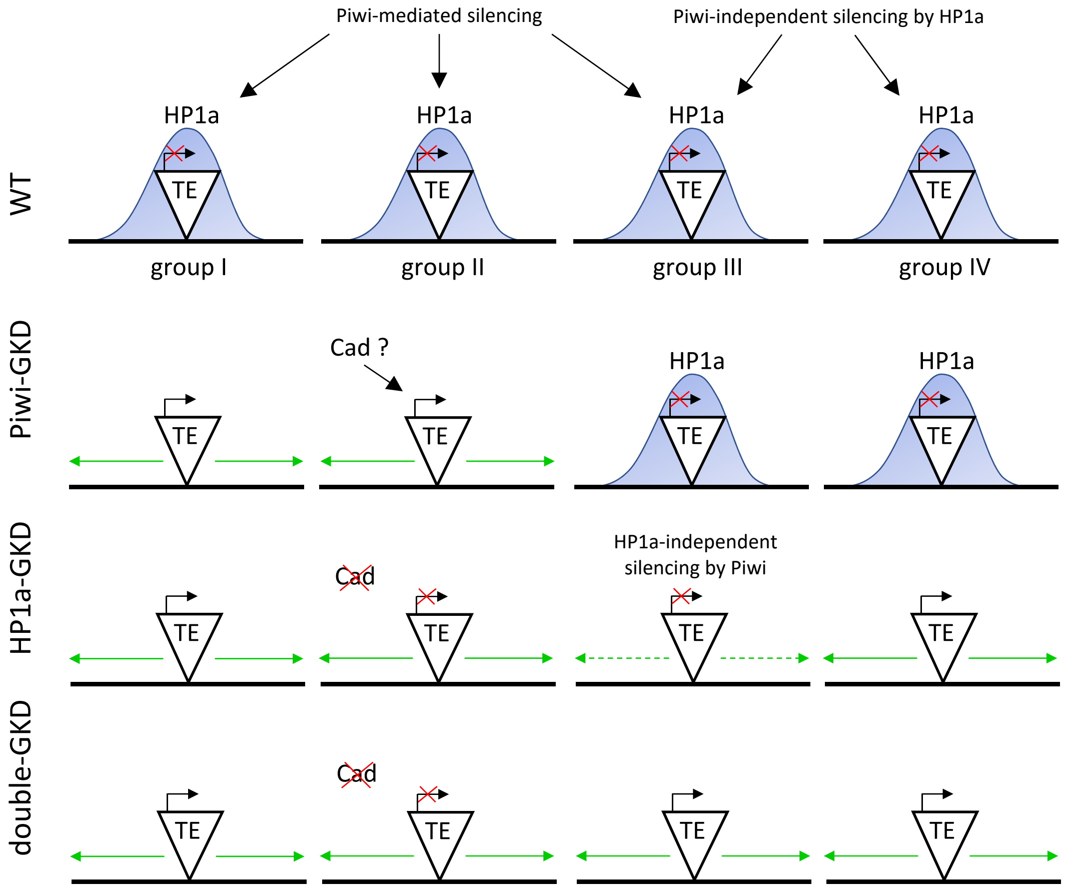

Figure S4. A hypothetical model to interpret different responses of TEs to Piwi, HP1a and double Piwi/HP1a germline knockdowns. Elevated transcription of genomic regions flanking TEs is indicated by green arrows. See text for details.
